# The Role of Animal Companions in the Bereavement Experiences of Australian Lesbians and Gay Men

**DOI:** 10.1177/00302228241237282

**Published:** 2024-02-28

**Authors:** Damien W. Riggs, Lefteris Patlamazoglou, Janette G. Simmonds, Tristan Snell

**Affiliations:** 1 College of Education, Psychology and Social Work, 1065 Flinders University, Adelaide, SA, Australia; 2 Faculty of Education, 2541 Monash University, Clayton, VIC, Australia

**Keywords:** animal companions, lesbian, gay, partner bereavement, loss

## Abstract

All too often, humans who experience the death of a partner are directed towards other humans for support, ignoring the important role that animal companions play in the lives of many humans. For lesbians and gay men specifically – whose grief may be disenfranchised – animal companions may play a particularly important role. This paper reports on a secondary analysis of interviews with 10 Australian lesbians or gay men who had lost a partner due to non-HIV related issues. Six of the participants spoke impromptu about the importance of animal companions following the death of a partner. Thematic analysis resulted in the development of three themes: (1) animals provide comfort and company, (2) animals serve as a reminder of partners, and (3) animals give people a reason to go on. The paper concludes by considering the importance of speaking about animals in the context of research and practice related to human bereavement.

## Introduction

Animal companions, and specifically domesticated animals who live in the home alongside humans, can play a significant role in the wellbeing of humans. Positive, caring relationships with animals have been found to result in reduced levels of distress for humans due to the soothing effects of animal companionship (Yorke, 2010), increased physical health, and a greater likelihood of positive social interactions with other humans (Wells, 2019). The benefits of animal companionship may be especially true for populations of humans who may otherwise be prone to increased stress, decreased physical health, and impaired social interactions with other humans. Older people are one group for whom animal companionship is likely to bring particular benefits (Garrity et al., 1989), as are people of diverse genders and sexualities (Taylor et al., 2019). The present paper focuses specifically on how animal companionship may be important to lesbians and gay men, including those of advanced age, who have experienced the death of a human partner.

To date, research on the role of animals in the bereavement experiences of humans has been minimal, and has focused almost exclusively on older heterosexual people. The earliest example of research in this area appears in the work of Bolin (1987), who surveyed 89 women living in the United States who had lost a husband, half of whom lived with a dog, and half of whom did not. Women who had a strong relationship with a dog adapted better to bereavement than did women who did not live with a dog. Bolin suggested that adaptation to bereavement for participants living with a dog may be due to having another living being to focus on in the face of the loss of a partner, and that the comfort provided by a dog may have had a therapeutic effect for the participants living with a dog.

More recent studies have similarly focused on heterosexual people’s relationships with animal companions following the death of a partner. Linn-Gust (2008) reports on a survey of 68 participants living in the United States, of whom 56 were women and 12 men, all of whom had lost a partner and lived with animals. Analysis of open-ended responses collected suggested that living with animals gave some participants hope in the context of loss, that having the physical presence of an animal helped assuage loneliness, and that the presence of an animal helped participants to stay active in the face of grief. Gabel (2013) conducted interviews with ten women who had lost a male partner, and who lived with animals in the United States. The findings note that while in the immediate time period following the death of a partner animals were not seen as a primary source of support, over time animals came to fill a void, including in terms of providing a link to a lost human partner, and in terms of recognizing grief behaviours exhibited by animals in the face of the death of a human. Most recently, Thompson and Kim (2023) interviewed 13 older heterosexual men who had lost their wives, and found that bonds with animals grew stronger after the loss, and animals helped the men to process the loss.

Research on lesbians and gay men suggests that to a certain degree bereavement experiences pertaining to a partner may be unique, due to the impact of disenfranchised grief (Doka, 1989). Stereotypes and prejudice directed towards lesbians and gay men, including in terms of the non-recognition of relationships, can mean that grief experienced following the death of a partner is not recognized by other people (Patlamazoglou et al., 2018). Research by McNutt (2014) with five gay men living in the United States who had lost a partner suggests that family members may often fail to recognize the loss as something to grieve, such as by expecting a bereaved person to move on quickly (because the relationship was not seen by others as significant), contesting inheritance rights, and excluding bereaved partners from funeral attendance. Similar findings in terms of lack of recognition of a loss by family members are evident in a study that included 17 gay men and ten lesbian women living in the United States (Nolan et al., 2019).

Given that disenfranchised grief may occur for lesbians and gay men, it is likely that animal companions may play a particularly important role, beyond that identified in previous research on bereaved heterosexual people. To date, much of the research on lesbians and gay men and animal companions has focused on older people (e.g., Muraco et al., 2018), but in only one instance has such research commented on the connection between animal companions and coping with the loss of a human partner. In terms of a broader focus on lesbians and gay men and animals, Putney (2014) interviewed 12 lesbian women living in the United States, exploring the significance of animal companions. Participants spoke about animals attenuating loneliness, giving a sense of purpose, and promoting a sense of acceptance through the perception that animals were non-judgmental. The latter was especially important in a broader societal context whereby older lesbian women had lived through time periods where anti-lesbian attitudes were highly salient. In terms of a specific focus on animals and bereavement, from the same study Putney (2013) has written about how animals may provide particular meaning to lesbian women in liminal periods, such as major life changes involving the loss of a partner.

Beyond the role of animal companions in coping with the loss of a human partner, a growing body of literature highlights the potentially unique meanings of animal companions to lesbians and gay men. A recent systematic review of this topic (Diaz Videla et al., 2023) reports that animal companions may be especially valued by lesbians and gay men given the broader context of heterosexism. Animal companions offer unconditional positive regard that may, at least to some extent, help to counter the negative effects of discrimination. Further, relationships with animal companions may help to foster human-human relationships, such as though walking animals or attending animal-related events. As a result, the literature summarized in the systematic review suggests that, through positive regard received from animal companions, lesbians and gay men may be uniquely attuned to their animal companions, and that this may specifically play out in terms of recognition of the marginalization that animals experience as a result of speciesism. Indeed, we might suggest that unconditional positive regard by animals provides recognition of the marginalization that lesbians and gay men experience, just as in turn lesbians and gay men may be more mindful of the harmful effects of speciesism on their animal companions.

As the limited research summarized above would suggest, animal companions can play an important role in the grief experiences of humans who lose a partner. To date the role of animal companions in the lives of lesbians and gay men who have lost a partner has been given very little attention, however the broader research on lesbians and gay men and loss, and lesbians and gay men and animal companions, suggests that animals may play an important role in processing grief that is disenfranchised, or by providing specific forms of positive regard that compensate for the fact of living in broader discriminatory societal contexts. The present paper sought to explore this topic in more detail by undertaking a secondary analysis of data collected as part of a broader study focused on the bereavement experiences of Australian lesbians and gay men.

## Method

### Participants

Participants from the primary study were four cisgender lesbians and six cisgender gay men (*N* = 10) who had experienced the death of a same- gender partner due to non-HIV-related reasons. Participants resided in Australia and were aged between 56 and 82 years. The number of years since their partners’ deaths ranged from 2 to 20 years, and the length of the relationships ranged from 6.5 to 45 years. The causes of death included cancer and heart and lung disease. For the secondary analysis reported in the present paper, the focus is on the six participants who spoke about bonds with animal companions following the death of a partner.

### Materials

Data were collected using individual, semi-structured interviews. The interviews were conducted in early 2017, before the postal vote that occurred in late 2017 to decide upon whether or not marriage equality would be legislated for. Interviews focused on changes experienced by participants after their partners’ deaths (e.g., ‘What changes did you experience due to your partner’s death?’), and responses from others to the death of a partner, including in terms of disenfranchised grief. These topics have been canvassed in previous publications, including reporting on disenfranchised grief experienced by our participants (Patlamazoglou et al., 2023). The average length of the initial interviews was 64.30 min Participants were not remunerated for their time.

### Procedure

Ethical clearance for the study was provided by the Monash University Human Ethics Committee before participant recruitment. Participants were recruited for the project using a purposive sampling strategy and those who were over 50 years old met the selection criteria of the present study. Invitations were distributed to LGBTQ organisations, community services and via social media. Once participants expressed interest in the study, they were then provided with an explanatory statement and a consent form, and the second author arranged a meeting for interview. Interviews were audio-recorded and transcribed verbatim to enable data analysis.

### Analytic Approach

Following Braun and Clarke (2023), the analysis reported in this paper adopts a ‘Big Q’ approach, referring to our interest in meaning-making practices among our participants in collaboration with us as researchers, rather than simply seeing interviews as a means to an end to collect a certain type of data (what Braun and Clark refer to as ‘small q’ approaches to qualitative research). In terms of our rationale for using Braun and Clarke’s (2006) approach to reflexive thematic analysis, again we echo Braun and Clarke (2023) in suggesting that the theoretical flexibility inherent to their approach was well suited to our interest in writing this paper. Specifically, our interest was to adopt a critical realist approach: one in which we could take as given our participants’ views along with our own views about what we saw on the video recordings in terms of the actions of animals, whilst also examining the broader social structures within which participant narratives are located.

For the purposes of the present article, we reviewed the entire primary data set, identifying instances where participants – unprompted – spoke about animal companions. Having collated this secondary data set, we then read and re-read the extracts repeatedly, and through this process developed themes that explained patterns identified by us as we viewed the data. We did not go into this process with a priori conceptualisations about how the data should or would look. The extracts included in the results below are indicative but not exhaustive of all extracts identified within the secondary data set.

## Results

From our analysis of the secondary data set, we developed three themes. While these to a certain extent echo the previous literature on heterosexual people’s bonds with animal companions following the death of a partner, our ‘Big Q’ approach allows us to consider more broadly how lesbians and gay men may uniquely experience bonds with animal companions following the death of a partner.

### Animals as Comfort and Company

In this first theme, participants spoke about the importance of the non-judgemental support offered by animal companions. Animals were often spoken about as providing both comfort and company following the death of a partner. For example:

I would talk to the cat and he’d listen. Well, the thing is, he was always there, before and after Nick died. So, just talking was like he knew what I was talking about. And I was calm when… when I talked to them, because… because I could only be calm talking to them (Brian).

Here Brian suggests that it was only when talking to his cat that he felt calm. In a world where the bereavement experiences of lesbians and gay men are often disenfranchised, having someone they could talk to and spend time with who would be non-judgemental was a hallmark of the role of animal companions for many of our participants. Vivien too spoke about the importance of the bond she shared with her dog:

I have a really good dog; I spend a lot of time at home alone, and I’m happy, you know. I’m alone, but it’s solitude I'm experiencing, not loneliness. Do I occasionally have loneliness? Sure! But I had that when Brooke was alive - who doesn’t? So, I actually have a pretty good life. As you saw, Deary (pointing at her dog) will bring her favourite toy to the door every time somebody walks in – she does it with everyone, even with me. So, this puts a smile on my face and takes my mind off things (Vivien).

For Vivien, Deary provides company that is valued, even if Vivien does not otherwise feel lonely. Nonetheless, despite saying she does not feel loneliness, Vivien suggests that she does need her mind taken off things (i.e., the death of Brooke), suggesting that Deary fulfills a unique role in her life. Other participants spoke about the role of animals during the end stages of life of their partner:

The cat which you just met, was very comforting, she was very good, you know; she, she’d be very comforting to Vince, she used to lie on him and purr for hours. And then that stopped, when he died... I don’t think grief ever disappears; I think it becomes part of you, and you live with it forever. But it certainly felt different not having Vince there for her to lie on (Carl).

Here Carl talks about a cat who spent time comforting his partner Vince, before his death. Importantly, Carl appears to acknowledge not only his own loss, but also the potential loss for the cat, who no longer had Vince there to lie on and comfort. This is important to note, as so often the focus on research about animals in the context of bereavement only focuses on human loss, ignoring the loss potentially experienced by animals. While it is not automatic that one disenfranchised group (i.e., lesbians and gay men) will recognise the loss experienced by another disenfranchised group (i.e., animals), there is certainly the potential for lesbians and gay men to be especially attuned to recognising how animals experience loss, and to be mindful of that in their own grieving.

### Animals as Reminders of a Partner

In this second theme, participants spoke about the important role of animal companions who served as a reminder of a lost partner, providing access to happy memories, as well as for some participants bringing further loss. For example, Josh spoke about happy memories shared with his partner and their dogs:

Kent and I would walk and play with the dogs together, and you know, share the responsibility as a couple. And I could see aspects of Kent in them (laughter)… Just, you know, the way he trained them (laughter). And they probably also missed Kent (Josh).

Here again, Josh acknowledges the loss potentially experienced by his dogs. He also speaks about being able to see aspects of his partner in the dogs. While clarifying that this pertains to training, we might also suggest a claim to kinship here, one that denotes the unique nature of relationships between lesbians and gay men and their animal companions, where animals are often considered part of the family. Other participants spoke about animals serving as reminders of partners, but that the death of an animal could compound the loss:

The last dog we had was Brooklyn, having him was, um, was a connection with Ignacio, you know, and so that helped… And then, you know, about 12 months after Ignacio’s death I have to have the dog put down because he had health issues and things. So, it was just another sort of cutting of the, you know, the ties, the bonds and things, you know (Trevor).

For Trevor, while Brooklyn provided a connection to Ignacio, Brooklyn’s death also exacerbated his sense of loss, having to mourn not only his partner, but then a year later mourn Brooklyn. Carl too spoke about the loss of an animal compounding the loss of a partner:

I’m always conscious of the fact that Vince was a big part of my life. And, you know, the thing that would have brought it up in the last few weeks was obviously the cat dying - that is so linked to Vince. And I'm sure I experienced the loss of the cat differently for that reason, because it’s another link gone (Carl).

Here Carl notes that the death of his cat was experienced in ways that might not have otherwise been the case. This is not to suggest that Carl would not have mourned the death of his cat, but rather to suggest that given the cat was a ‘link’ to Vince, their death meant that they were no longer present to serve as a reminder of Vince.

### Animals as a Reason to Keep Going

In this final theme participants spoke about animal companions giving them a reason to live after the death of a partner. Brian spoke the most about this, emphasising that having an animal was one of a number of reasons why he didn’t just ‘curl up into a ball’:

Having the animal was probably the third element that gave me a reason to continue coping. Uhm... So, you know, in terms of work, my responsibilities as a child with an elderly parent and my responsibilities as a pet owner, (laughter) kind of became... It kept me going. You know, you've got these things that were, you know, there’s a... I was responsible for a level of care and a level of dependency that meant that I didn’t kind of curl up into a ball and, in some ways, wallow in my own misery. Which, I suspect, is a temptation, in many cases, but having those outlets, those avenues, kind of, I guess, helped in terms of those coping mechanisms (Brian).

In this extract we would note the laughter when Brian mentions animal-related responsibilities, just as Josh laughed in the previous theme when he commented on seeing something of his partner in his dogs. Given the interview questions did not ask about animals, this laughter may signal the non-normative orientation of including animals in a conversation about coping following the loss of a partner. While, as summarised in the introduction to this paper, animals can be an important source of support, this does not mean that our love for animals is not also disenfranchised: that some humans may discount the importance of animal companionship following the death of a partner. Similar to Brian, Caroline suggested that walking her dogs stopped her being ‘in my pyjamas all day’:

Like, I walk the dogs every day, which is a meditation. I think I know after these 20 years that’s it’s so easy for emotions to become quite negative that I believe I need to maintain a bit of a routine. And these walks have helped me keep up the routine; otherwise, I would be like in my pyjamas all day (Caroline).

For Caroline, walking her dogs provides a routine. Following the death of a partner, many people experience a loss or destabilisation of routine. Animals can thus help humans to maintain routines in the face of grief, or indeed to establish new routines. Indeed, as Caroline notes, walking the dogs ‘is a meditation’, which accords more significance to this activity than simply a routine. Rather, it potentially provides a pathway to healing.

## Discussion

In this paper we have provided a secondary analysis of interviews where Australian lesbians and gay men spoke about non-HIV related loss of a partner, and where a majority brought up, impromptu, the role of their animal companions in coping with their loss. While to a certain extent our findings echo previous research on heterosexual people and the role of animals in coping with the loss of a partner (e.g., Gabel, 2013; Linn-Gust, 2008; Thompson & Kim, 2023), our findings also suggest potential examples of how animals may play a unique role in the lives of bereaved lesbians and gay men.

Firstly, while not explicitly stated in the excerpts included in this paper, our participants most certainly spoke about their grief being disenfranchised by family and other people throughout their interviews (Patlamazoglou et al., 2023). That, by contrast, animal companions appeared to provide recognition and comfort in the face of disenfranchisement due to heteronormativity, would suggest a specific role played by animal companions for bereaved lesbians and gay men. Further, when speaking about their animal companions, some of our participants appeared to orient to an idea of kinship with animals, echoing previous research (e.g., Riggs et al., 2021). There are certainly long histories of lesbians and gay men engaging in animal-related advocacy, and in forming chosen families that include animals. Indeed, Monk (2016) explores the phenomenon of lesbians and gay men including provisions for their animal companions in their wills, or donating their assets to animal charities. That animals may be considered kin by some lesbians and gay men, and hence may play an important supporting role during grief, again highlights the distinctive role of animals in experiences of bereavement for lesbians and gay men.

Further to the potentially specific role that animal companions may play in the bereavement experiences of lesbians and gay men, is the possible recognition that lesbians and gay men may give to animals’ experiences of loss. Certainly, many humans may show kindness to animals in the face of loss, but there is the possibility that lesbians and gay men – through their own experiences of disenfranchisement – may be particularly attuned to the loss experienced by animals, and may in turn provide support to their animal companions. Again, previous research has found that lesbians in particular may be sympathetic to loss experienced by their animal companions: both the loss of a human, and the loss of another animal (Riggs et al., 2021).

### Limitations

While we believe that there are some major strengths of the findings reported in this paper, we must also acknowledge limitations. This was a secondary analysis of comments provided by our participants unprompted. While this is a strength – that participants spoke about animals as sources of support – it is also a limitation, in that we did not ask about animals specifically. We also acknowledge the limitations to the sample, both in size, but also in terms of being restricted to cisgender lesbians and gay men. Research suggests more broadly that bisexual, trans, and queer people also experience unique relationships with animal companions, relationships that are likely to bring meaning following the death of a partner (Riggs et al., 2021). Further, while the focus on non-HIV related death was purposive (given the pronounced focus on HIV-related deaths in the literature), research nonetheless suggests that animals may be especially important to people who live with HIV (Grierson et al., 2023, and thus potentially for their partners if a death occurs. Future research will likely benefit from a closer focus on the experiences of partners who are bereaved due to HIV in terms of animal companionship. Finally, we note the possibility that, since the introduction of marriage equality in Australia, it may be the case that the grief experiences of lesbians and gay men may be less likely to be disenfranchised. Of course the introduction of a law does not necessarily translate to social change, and this deserves further attention in future research in terms of the bereavement experiences of lesbians and gay men in Australia.

### Implications

In terms of research, the research reported in this paper would suggest the importance of asking about animal companions when speaking to or surveying people about bereavement, and here specifically with regard to lesbians and gay men. This should include not simply asking about the role of animals in helping humans to process grief, but also asking about their animal’s experiences of loss. Honing in on the mechanisms by which animals help and are helped, and how this plays out in specific ways for lesbians and gay men, thus constitutes an important focus for future research. This might include conducting interviews where animals are present, so as to be able to observe the relationship, and actively prompt all parties to engage with conversations about the relationship and its role in healing from loss.

In terms of practice, it is all too common for people to mention animals in sessions with health professionals, only for animals as a topic or source of support not to be actively taken up (Riggs et al., 2023). It is thus important that health professionals are mindful of the often important role of animals in the lives of many people, but here especially lesbians and gay men, and to actively and purposively ask about animals. As some of our participants suggested, this may include the role of animals in helping humans maintain or establish routines following bereavement. But it may also include conversations about how the later death of an animal companion may compound human-related experiences of grief, or how animals who continue to live following the death of a human partner may be experienced as a burden in the face of grief. Health professionals who are mindful of the many challenges that humans may face in their relationships with animals and in the context of bereavement are those most likely to help to find pathways to healing.

In conclusion, this paper highlights the distinctive and important role of animal companions in the lives of lesbians and gay men who experience the death of a partner. The findings also provide an impetus for further research, and provides an initial template for thinking through what the findings and future research mean for practice with humans who experience bereavement. Yet beyond human-related implications, the findings reported in this paper take a necessary first step towards recognition of animal experiences of loss in the context of lesbian and gay lives, a focus that is vitally important given the need to move beyond anthropocentric understandings of loss.
